# Myocardial Infarction in a Young Cocaine User with Atrial Myxoma: What is the Central Cause?

**DOI:** 10.7759/cureus.2325

**Published:** 2018-03-14

**Authors:** Eduardo L Santos, Renata M Gonçalves, Arthur C Holanda, Vinícius O Nogueira, Luís C Correia

**Affiliations:** 1 Department of Cardiology, UFPE; 2 Department of Cardiology, Hospital De Base Doutor Ary Pinheiro; 3 Medical School, UFPE; 4 Department of Cardiology, Hospital E Pronto Socorro João Paulo Ii; 5 Department of Cardiology, Hospital São Rafael

**Keywords:** myocardial infarction, cocaine, heart neoplasms

## Abstract

The differential diagnosis of myocardial infarction in patients with a number of potential causes can be challenging, especially when there is no angiographic evidence of coronary obstruction. We describe a case of extensive anterior ST-elevation myocardial infarction in a young male smoker who had a left atrial myxoma and a history of cocaine use 10 hours before the event. Clinical reasoning during a myocardial infarction investigation should be oriented by the probability of the causal relationship between the ischemic event and each factor present in the clinical context.

## Introduction

The physiopathology of myocardial infarction (MI) is associated majorly with the rupture of an atherosclerotic plaque in the coronary circulation, coronary embolism and coronary vasospasm being among the less common causes [1]. Although an etiological diagnosis is usually reached easily, a differential diagnosis in patients with a number of potential causes of the ischemic event can be challenging, especially when there is no angiographic evidence of coronary obstruction. In this report, we describe a case of extensive anterior ST-elevation myocardial infarction (STEMI) in a young male smoker who had a left atrial myxoma and a history of cocaine use 10 hours before the event.

## Case presentation

**Figure 1:**
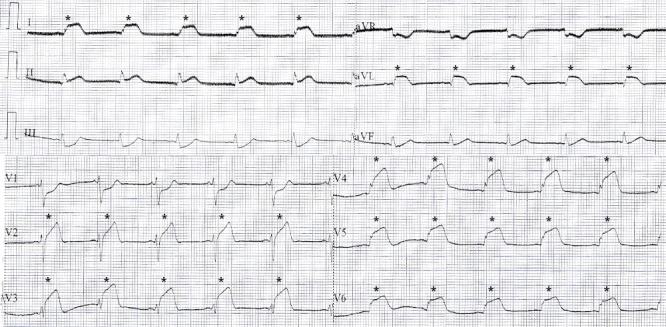
STEMI at electrocardiography ST-elevation compromising V2 to V6, DI, and aVL derivations (*). STEMI: ST-elevation myocardial infarction

A 42-year-old male was admitted with high-intensity constrictive precordial pain, without irradiations, associated with diaphoresis, dyspnea, and cutaneous-mucous paleness after an important emotional stress. The patient referred current tobacco and amphetamine use, a history of alcoholism (abstainer for 10 years), and the use of cocaine around 10 hours before the event. A physical exam identified bilateral pulmonary rales, with a clinical picture compatible with cardiogenic acute pulmonary edema, which was corroborated by the presence of a bilateral infiltrate at chest radiography. The electrocardiogram (ECG) demonstrated an extensive anterior ST-elevation myocardial infarction (STEMI), as shown in Figure 1. The curve of myocardial necrosis markers during admission was consistent with the electrocardiographic diagnosis.

The patient was submitted to thrombolytic therapy with tecneteplase. Despite the progressive improvement of the precordial pain, ST-elevation had not fallen under 50% of its original amplitude 90 minutes after the administration of the fibrinolytic. Thus, he was referred for rescue cardiac angiography, which revealed flow reduction (TIMI 2) in the left anterior descending artery (LAD) territory, without significant coronary obstructions. During an investigation at the emergency department, the patient also underwent a head computed tomography (CT), which was normal, and an abdomen ultrasonography (US), through which hepatic steatosis grade I and left pleural effusion were identified.

**Figure 2:**
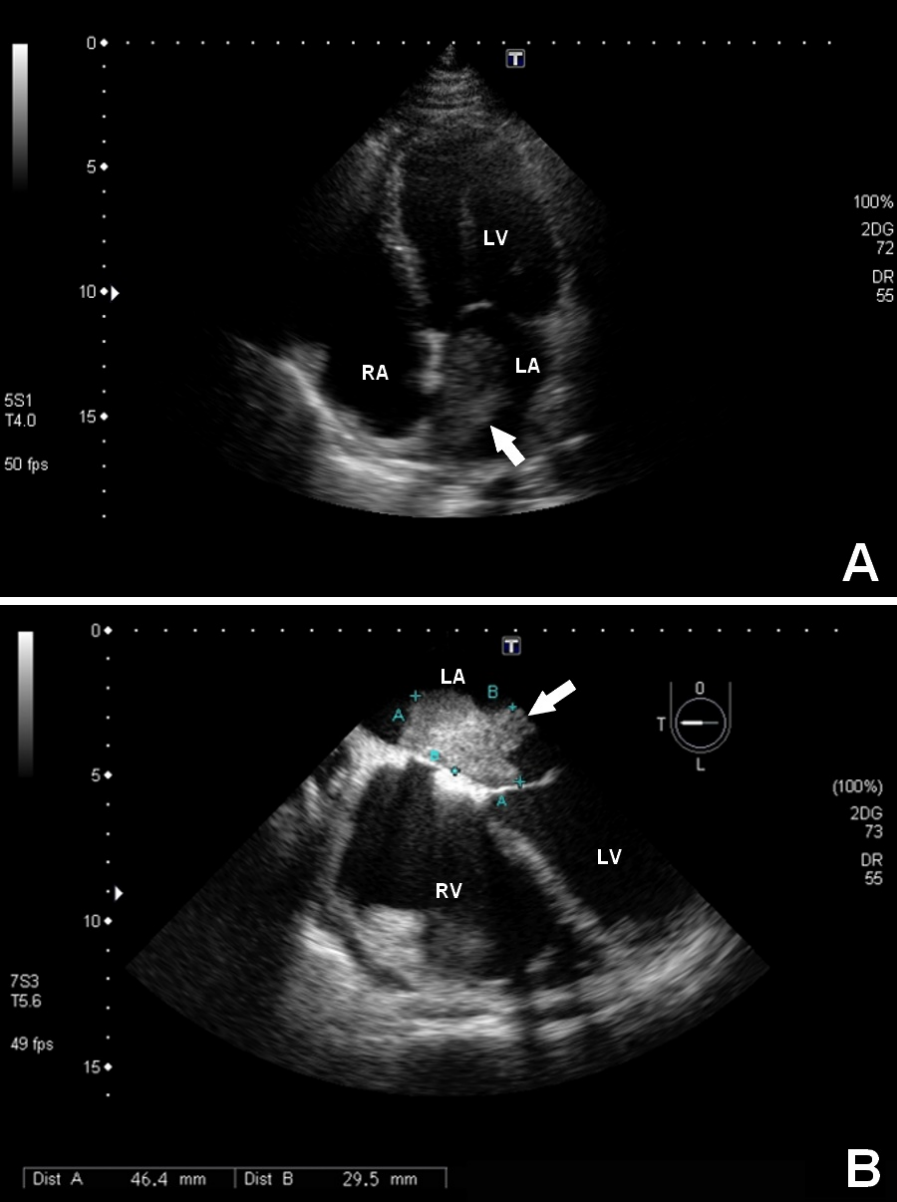
Left atrial myxoma at echocardiography (A) Apical four-chamber view of transthoracic echocardiography showing a large, heterogeneous, irregular, hyperechoic mass inside the left atrium (arrow) (47x30mm). (B) Transesophagic echocardiography showing the myxoma fixed in the interatrial septal wall (arrow) (46.4x29.5mm). LA - left atrium, LV - left ventricle, RA - right atrium, RV - right ventricle.

A transthoracic echocardiogram (TTE) was performed the day following the admission. It revealed pseudonormal diastolic dysfunction and apical/lateral anteroseptal akinesia, as well as a pedunculated mass adhered to the left atrium septal wall, measuring 4.7x3.0 centimeters, consistent with atrial myxoma (Figure 2A). The etiologic investigation continued at the clinical ward of the service, with a transesophagic echocardiogram (TEE) better showing the mass adhered to the interatrial septal wall (Figure 2B, Video 1, and Video 2).

**Video 1:** Left atrial myxoma at transesophagic echocardiography Transesophagic echocardiography showing a multilobulated, highly mobile left atrial myxoma.

**Video 2:** Left atrial myxoma at transesophagic echocardiography Besides the myxoma, there is an imaging artifact inside the right ventricle, which was not visualized in other projections.

## Discussion

Our patient presented at least three possible causes for his STEMI, none of them corresponding to the usual clinical context of myocardial infarction. First, the STEMI could be secondary to coronary embolism from his left atrial myxoma. Second, it could result from a coronary spasm as a late consequence of his acute history of cocaine use. Third, it could be an early case of MI due to atherosclerotic disease, as he had tabagism as an important risk factor. Reaching the final diagnosis based only on the clinical investigation is unlikely, especially because no evidence of coronary artery disease or coronary obstruction was identified during cardiac angiography. Therefore, a determination of the cause relies mainly on the analysis of the probability of each of the above-mentioned situations.

Although systemic embolic events are very common in left atrial myxomas − with frequencies up to 65.0% − the coronary system is affected in only 0.06% of these events [2]. The low frequency of coronary artery embolism is explained by the right angulation of the coronary arteries with the aorta, by the protection provided by the aortic valve leaflets during systole, and by the small opening diameter of the coronary arteries [3]. Our patient presented a highly mobile myxoma image, with a markedly irregular surface and several small mobile images adhered to its surface, characteristics that indicate a high probability of embolization [4]. However, as he did not present other systemic embolisms, the probability of cardiac embolization due to a highly embolic myxoma decreases. On the other hand, the non-visualization of coronary occlusion by cardiac angiography does not rule out the possibility of myxoma emboli in the coronary circulation, as it occurred in 40% of the 19 cases reported in the last 10 years. This finding would be a result of the response to fibrinolytic therapy or spontaneous degradation of emboli with a major gelatinous stromal component [5].

Cocaine is a well-known inducer of myocardial ischemia, corresponding to 1% of the cases of MI, and its pathophysiology is related to the association between increased oxygen demand, coronary artery vasospasm, and platelet activation promoted by the drug [6]. Normal coronary arteries are very common in cocaine-induced MI, being identified in 38% of the 92 patients analyzed by Minor et al. [7]. In our patient, an argument against the causal link between cocaine and MI is the long time-span between events. While here, the cocaine use occurred 10 hours before the MI, the literature points that one-fourth of cocaine-induced MI cases occur during the first hour after exposition − when the risk is 24 times greater than the baseline [6], with two-thirds occurring within three hours of cocaine ingestion [8]. However, some studies have shown that the interval between cocaine and MI can last several hours or even a few days, due to the persistence of cocaine metabolites in the circulation [8].

Adults younger than 45 years correspond to 10% of all the cases of MI [9]. Smoking is the most common risk factor for early MI, with smokers representing a percentage as high as 91% of these patients [9]. In smokers with normal coronary arteries, MI can be caused by temporal coronary occlusion due to thrombus formation − associated with an induced enhancement in platelet aggregation − or coronary artery spasm [10]. Although not an acute inducer of vasoconstriction in patients with normal coronary arteries, smoking can also impair endothelial-dependent vasodilatation, increasing the susceptibility of vasospasm by agents such as alcohol and cocaine [10].

Our patient presented an atypical clinical context for his MI, regardless of which of the causes was indeed responsible for it. In fact, it is possible that more than one, or even all three, were involved. Tobacco use − depending on duration and intensity, both unknown in this case − could have chronically originated the substrate for a late cocaine-induced coronary spasm, through the impairment of vasodilatation or the development of atherosclerotic plaques. In the latter case, the normal angiographic appearance of the coronary arteries could result from the association between atherosclerosis, medial atrophy, and vessel wall dilatation [10]. Although atrial myxoma embolization was less likely involved, the intracardiac mass could have reduced blood supply to the coronary arteries in a situation of high oxygen demand (i.e., cocaine use), as its presence can also result in atrioventricular valve obstruction [2].

Among art movements, Impressionism stands out for its loosened brushwork, vibrating colors, and imprecise and non-delimited traits, with focus directed away from clarity and detail. In clinical judgment, impressionist thinking can be harmful (i.e., establishing causality links relying only on how overwhelming a diagnosis is), and it should never overcome probability. In the case described here, even though a confirmation of the cause cannot be achieved, probabilistic thinking favors the history of cocaine use as the most responsible for the ischemic event. While 0.039% of the patients with a left atrial myxoma embolize to coronary circulation [2], MI has been shown to occur in 0.7% to 6.0% of patients who present precordial pain and a history of cocaine use in the emergency department [8]. Considering the lowest percentage, even if cocaine ingestion had occurred more than 24 hours before the event, the number would be 0.084% [8].

## Conclusions

The case described in this report exemplifies how challenging the investigation of MI causes can become. Clinical reasoning in such cases should be oriented by the probability of the causal relationship between the ischemic event and each factor present in the clinical context. In our patient, it is most probable that cocaine use played the major role, even though the real etiology is difficult to confirm and other factors, including the presence of a left atrial myxoma, could have been involved in the development of MI.
